# Johanson-Blizzard Syndrome: A Case Report From Bahrain With a Literature Review

**DOI:** 10.7759/cureus.55969

**Published:** 2024-03-11

**Authors:** Hasan M Isa, Zainab A Khudhair, Kawthar M Abdulla, Zahra A Idrees, Maryam Y Busehail, Zainab A Jawad

**Affiliations:** 1 Department of Pediatrics, Arabian Gulf University, Manama, BHR; 2 Department of Pediatrics, Salmaniya Medical Complex, Manama, BHR; 3 Department of Otolaryngology, Salmaniya Medical Complex, Manama, BHR

**Keywords:** cutis aplasia, sensorineural deafness, exocrine pancreatitis insufficiency, hypoplastic alae nasi, johanson-blizzard syndrome

## Abstract

Johanson-Blizzard syndrome (JBS) is a rare hereditary autosomal recessive disorder caused by a mutation in the ubiquitin protein ligase E3 component n-recognin 1 (UBR1) gene. This syndrome is characterized by the following typical clinical features: hypoplasia or aplasia of the alae nasi, congenital scalp defects, sensorineural hearing loss, hypothyroidism, growth retardation, psychomotor retardation, imperforate anus, genitourinary anomalies, and atypical hair patterns. Here, we describe a case of a 12-year-old girl with JBS of consanguineous parents. During the last trimester of pregnancy, a congenital abnormality affecting the nose was detected. Immediately after birth, the clinical examination revealed dysmorphic features in the form of hypoplastic alae nasi, microcephaly, mild hypotelorism, and cutis aplasia on the scalp. The genetic testing of the patient showed a novel sequence change mutation of the UBR1 gene (1bp duplication causing a frameshift), while both parents were carriers for this mutation. Moreover, a diagnosis of pancreatic insufficiency and subclinical hypothyroidism was made based on clinical presentation and laboratory results. The patient was started on pancreatic enzyme replacement therapy and fat-soluble vitamins, minerals, and antioxidant syrup. Further assessment revealed hypotonia, growth impairment, delay in reaching developmental milestones, and bilateral profound sensorineural hearing loss, which was managed with bilateral cochlear implantation. In addition, the patient underwent multiple craniofacial reconstructive surgeries. This case report highlights the importance of early diagnosis and multidisciplinary care of patients with JBS.

## Introduction

Johanson-Blizzard syndrome (JBS) is a very rare genetic disorder. Ann J. Johanson and Robert M. Blizzard are the pediatricians who reported and described this syndrome in 1971 in three unrelated girls with distinctive physical dysmorphism [1,2]. It was genetically unknown until 2005 when Zenker et al. found that this syndrome is inherited as autosomal recessive and caused by a mutation in the ubiquitin-protein ligase E3 component n-recognin 1 (UBR1) gene located on chromosome 15q15-21 [3]. UBR1 is responsible for encoding one to four functionally overlapping E3 ubiquitin ligases of the N-end rule pathway [3]. It is yet unknown what specific pathophysiological relationship exists between altered protein degradation and the clinical abnormalities seen in JBS [4]. JBS is a collection of abnormalities primarily affecting midline structures that develop between weeks six and eight of pregnancy [5].

This syndrome affects both males and females equally [4]. JBS is characterized by the following typical clinical features: hypoplasia or aplasia of the alae nasi, congenital scalp defects, sensorineural hearing loss, hypothyroidism, growth and mental retardation, imperforate anus, genitourinary anomalies, and dental problems [2]. Moreover, pancreatic insufficiency is a consistent feature of this syndrome [6]. Additional features such as cardiac abnormalities, microcephaly, atypical hair patterns, and short stature have been reported [6]. Patients with this syndrome usually have moderate-to-severe mental retardation, yet normal intellect can also occur [7].

Globally, there are less than 100 documented patients in the literature [8]. In Saudi Arabia, a neighboring country to Bahrain, the first case with JBS was reported in 2008 [9]. However, this syndrome was not previously reported in Bahrain. Hence, we are reporting the first case in the Kingdom of Bahrain along with a literature review.

## Case presentation

**Figure 1:**
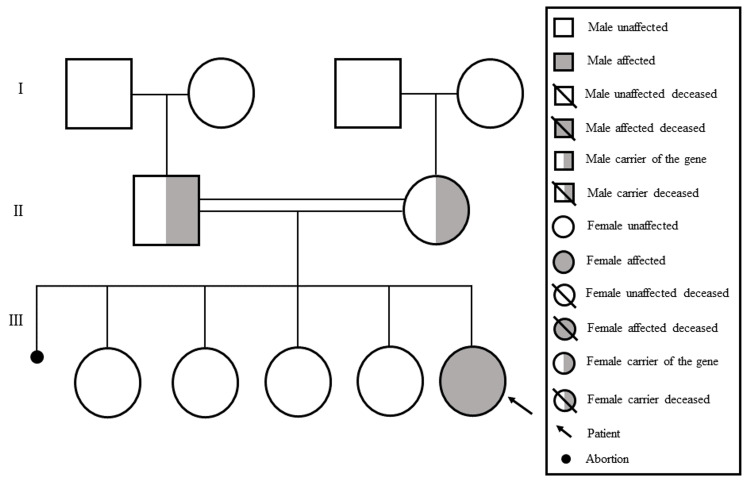
Family pedigree of the child with Johanson-Blizzard syndrome. Image credits: Hasan M. Isa, Zainab A. Khudhair, Kawthar M. Abdulla, Zahra A. Idrees, Maryam Y. Busehail, Zainab A. Jawad.

A 12-year-old Bahraini girl was born at Salmaniya Medical Complex at full term through vaginal delivery, with Apgar scores of 8 and 9 at 1 and 5 minutes, respectively. A prenatal sonography performed in the last trimester showed a congenital abnormality affecting the nose. The mother’s age during pregnancy was 38 years. The patient was the fifth child of consanguineous parents who had four other healthy daughters and had one abortion (Figure 1).

**Figure 2:**
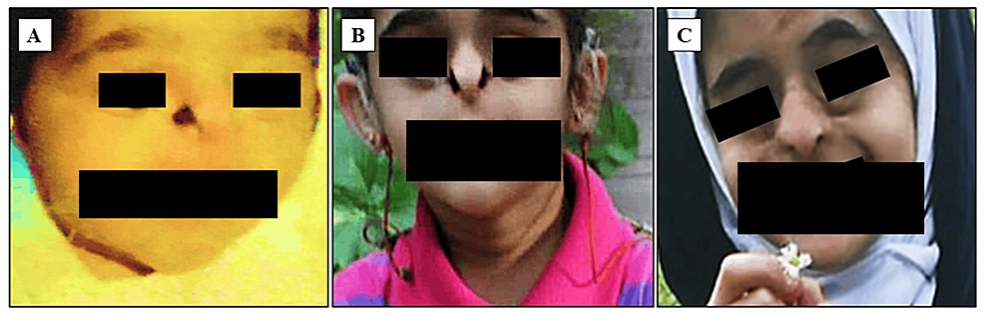
A child with Johanson-Blizzard syndrome and dysmorphic features. (A) Soon after delivery; (B) before the nasal plastic reconstructive surgery (wearing bilateral external sound processors after bilateral cochlear implantation); (C) after the nasal plastic reconstructive surgery (started at the age of six years).

After delivery with a birth weight of 2,640 g, the patient’s physical examination revealed dysmorphic features in the form of hypoplastic alae nasi, microcephaly, mild hypotelorism, and cutis aplasia on the scalp (Figure 2A). 

Eye examination was normal. Other systemic examinations were unremarkable. The patient underwent extensive evaluation by multiple medical teams, including specialists in neonatology, otorhinolaryngology, plastic surgery, endocrinology, radiology, and genetics. Laboratory results showed a hemoglobin level of 9.2 g/dL with low indices. Detailed laboratory findings of the patient are shown in Table 1. 

**Table 1:** Laboratory results of a patient with Johanson-Blizzard syndrome. MCV, mean cell volume; MCH, mean cell hemoglobin; NR, no record; RBCs, red blood cells; MPV, mean platelet volume; WBCs, white blood cells

Laboratory test	Normal range	Patient age (years)					
		0.2	2.4	2.6	6.0	7.0	12.0
Hemoglobin (g/dL)	12-14.5	9.2	10.5	10.5	9.7	9.9	8.2
Hematocrit (%)	33-45	28.0	33.0	32.0	35.4	33.3	28.9
MCV (fL)	82-97	73.5	64.6	63.4	67.5	60.1	57.9
MCH (pg)	27-33	23.6	20.7	NR	18.5	17.8	16.4
RBCs (cells/µL)	3.9-5.2	3.9	5.1	5.1	5.2	5.6	4.9
Platelets (×10⁹/L)	140-440	509	342	229	360	378	299
MPV (fL)	7.8-11	NR	7.7	7.3	8.0	6.5	9.1
WBCs (×10⁹/L)	3.6-9.6	16.3	6.2	5.2	3.8	4.0	4.0
Neutrophils (%)	42.2-75.2	NR	NR	NR	37.3	34.6	40.4
Lymphocytes (%)	20.5-51.1	69.0	62.0	55.0	55.5	56.4	44.0
Monocytes (%)	1.7-9.3	11.0	8.0	10.0	5.1	5.3	13.5
Eosinophils (%)	1-6	2.0	3.0	3.0	2.0	3.0	2.0
Basophils (%)	0-2	0.0	0.0	NR	0.2	0.7	0.3
Iron (µmol)	9.0-30.4	NR	NR	NR	6.0	9.0	3.0
Transferrin (g/dL)	2.5-3.8	NR	NR	NR	3.9	3.9	3.7
Transferrin saturation (%)	15-33	NR	NR	NR	6.0	9.0	3.0
Vitamin D (nmol/L)	≥50	NR	NR	NR	77.0	NR	53.0

**Figure 3:**
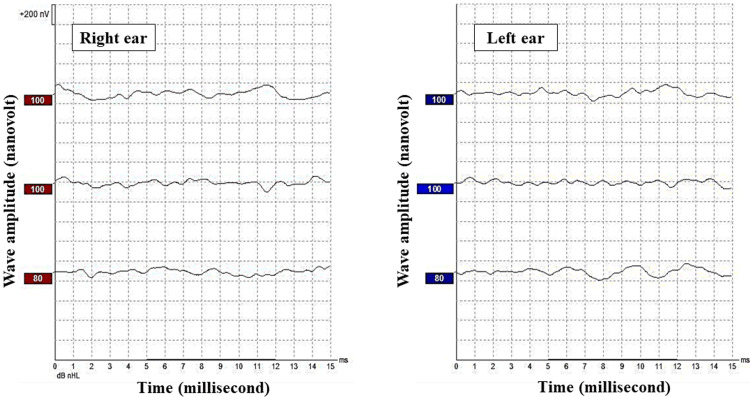
A brainstem auditory-evoked response trace of a child with Johanson-Blizzard syndrome showing no response at maximum auditory stimulation (100 dB nHL) in both ears, which indicates a bilateral profound hearing loss. nHL, normal hearing level

Subclinical hypothyroidism was initially diagnosed, but the thyroid-stimulating hormone level improved spontaneously from 10.6 to 6.7 mIU/L (normal level <6 mIU/L). Serum electrolytes, calcium, phosphorus, urea, creatinine, liver function tests, and glucose-6-phosphate dehydrogenase levels were all within the normal range. A stool fat globules test was positive, but fecal tryptic activity was normal with a dilution of 1:3200, the patient is considered to have pancreatic insufficiency if fecal tryptic activity dilution is less than 1:50. Abdominal ultrasound (US), echocardiogram, and brain magnetic resonance imaging (MRI) were all reported to be normal. Hearing assessment was done using an auditory brainstem response test, which showed no response up to 100 dB normal hearing level (nHL) in both ears (average hearing threshold for healthy newborns: 20-40 dB nHL) (Figure 3). 

Given dysmorphism, the genetic specialist requested genetic testing, and blood samples of the child and both parents were sent to Germany. The microarray study showed a novel sequence change mutation of the *UBR1 *gene (1 bp duplication causing a frameshift). Both parents were heterozygous carriers of this mutation. This confirmed the diagnosis of JBS.

**Figure 4:**
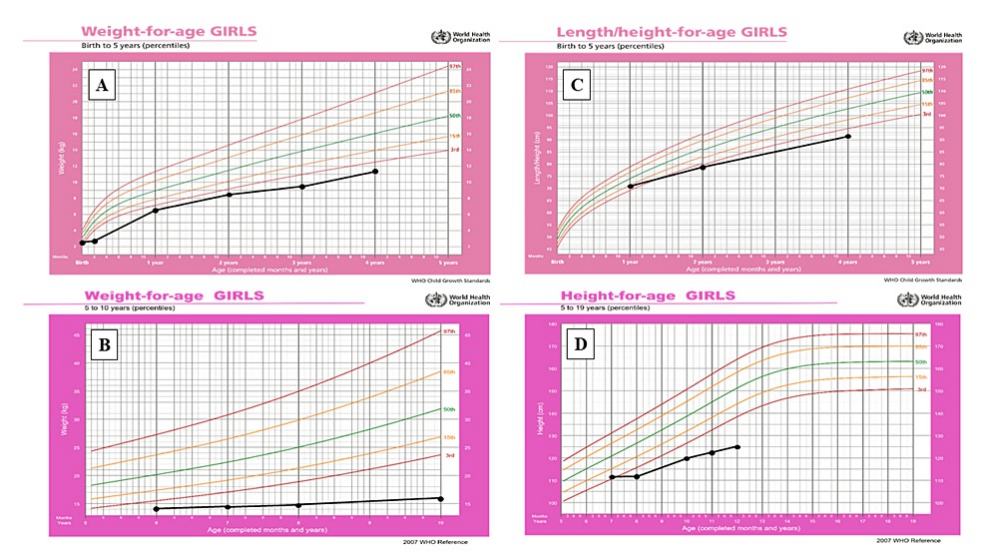
World Health Organization growth charts showing the anthropometric parameters of a child with Johanson-Blizzard syndrome. (A) and (B) Weight-for-age measurements from birth to five years and five to 10 years, respectively; (C) and (D) length/height-for-age measurements from birth to five years and five to 10 years, respectively.

Shortly after delivery, the patient commenced combined breast and formula milk feeding. The parents observed abdominal distention, oily foul-smelling stools, and nonbilious, nonprojectile vomiting after each feeding. Moreover, delayed growth was also detected and manifested as poor weight and height gains (Figure 4). 

Based on clinical presentation and laboratory results, a diagnosis of pancreatic insufficiency was confirmed. The patient was started on pancreatic enzyme replacement therapy (Creon 1 capsule per kilogram), fat-soluble vitamins, minerals, and antioxidant syrup along with dietary advice to transition to a specialized anti-regurgitation milk formula. Thereafter, the patient required regular follow-up and management by a pediatric gastroenterology consultant. However, the patient experienced a delay in reaching developmental milestones as she started first rolling over at the age of one year and walking at the age of one and a half years. Furthermore, she had hypotonia, but her motor functions were normal.

**Figure 5:**
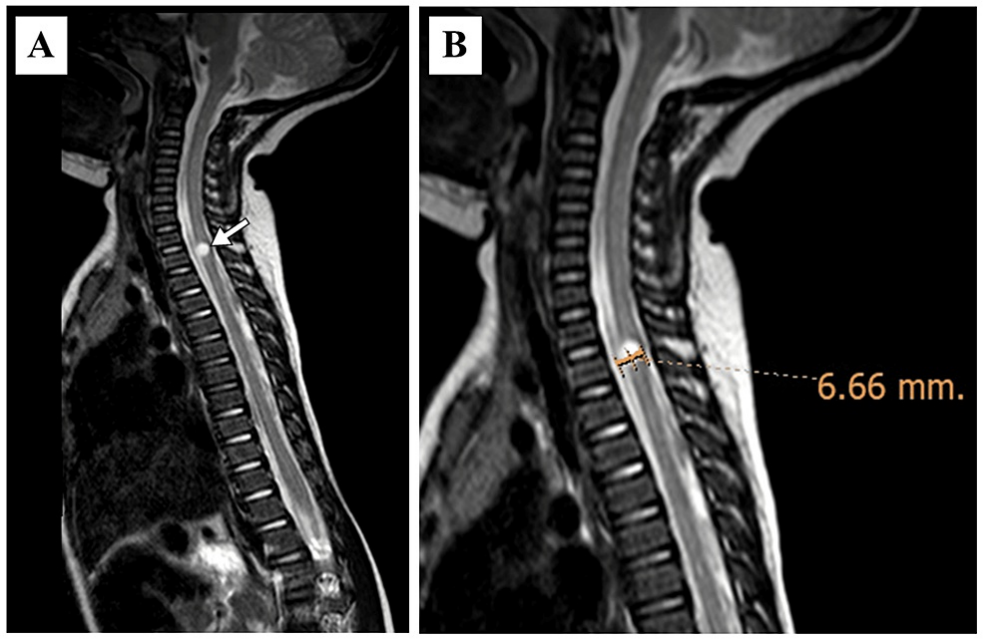
A sagittal T2-weighted magnetic resonance imaging of the spine of a child with Johanson-Blizzard syndrome. (A) A hyperintense cystic lesion at the anterior aspect of the spinal cord at the second thoracic vertebral level (arrow), most likely representing a syringohydromyelia; (B) a close-up image demonstrates the size of the cystic lesion.

At the age of two years, serial imaging was obtained. An MRI of the spine was conducted as a part of the brain and internal auditory meatus (IAM) protocol, which revealed an incidental, eccentrically placed cystic lesion at the anterior aspect of the spinal cord at the level of the second thoracic vertebra, which most likely represented a syringohydromyelia. However, further clinical correlation to assess for upper limb muscle tone and follow-up with an MRI was advised (Figure 5). 

At the age of three years, the patient underwent sequential bilateral cochlear implantation. Postoperatively, aural rehabilitation with the speech therapist was provided, and she had to wear bilateral external sound processors (Figure 2B).

At the age of six years, the patient started to undergo nose and palpebral reconstructive surgeries in France and Bahrain (Figure 2C).

**Figure 6:**
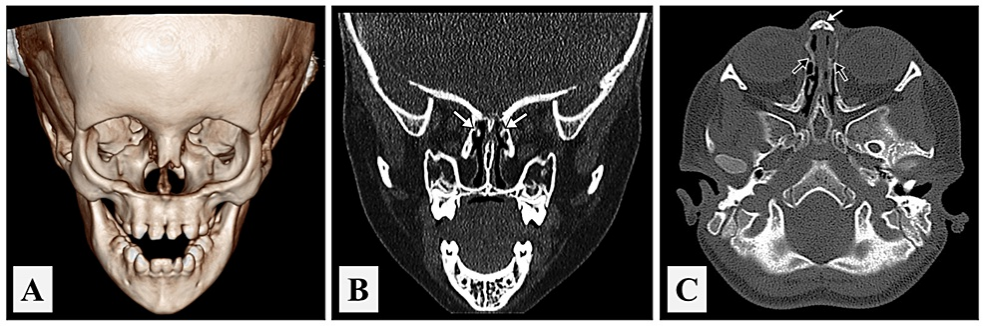
A computed tomography of a child with Johanson-Blizzard syndrome. (A) A three-dimensional computed tomography scan of facial bones; (B) a coronal computed tomography scan of the nose and paranasal sinuses showing aplasia of maxillary sinuses, small middle turbinates (arrows), and absent inferior turbinates; (C) an axial computed tomography scan of the nose and paranasal sinuses showing short nasal bones (white arrow), absent frontal processes of maxillary bones, and loss of bony boundaries of nasolacrimal ducts (black arrows).

At the age of eight years, the patient experienced orbital edema for which a computed tomography (CT) scan for the orbit and sinuses was conducted. The scan revealed bilateral bony defects at anteromedial orbital aspects due to the missed frontal process of maxillary bones, lacrimal bones, and short nasal bones. Moreover, there was a loss of the bony boundaries of the nasolacrimal ducts, which looked narrow, and a nonvisualized inferior nasal turbinate with a small-sized middle nasal turbinate was detected (Figure 6).

Accordingly, she underwent a dacryocystorhinostomy operation. Thereafter, the patient required several hospitalizations for nose, palpebral, and ear surgeries until she reached the age of 12 years. The next surgery will be performed when she reaches the age of 14 years.

Currently, the patient has reached the age of 12 years and three months. She has good social skills and attends school regularly, but she is two years delayed from what she was supposed to achieve. Besides, she is receiving follow-ups at pediatric gastrointestinal, dietetic, and plastic surgery clinics, and she continues to attend speech therapy sessions. She is doing well apart from having dental problems, for which she is wearing a pediatric dental bridge. She is disciplined in taking pancreatic insufficiency medications, which include pancreatic enzyme replacement therapy (Creon 1 capsule per kilogram) and fat-soluble vitamins, minerals, and antioxidant syrup. Despite adhering well to medical therapy, her growth parameters remain below the 3rd percentile, indicating the need for ongoing monitoring and the implementation of appropriate dietary measures.

## Discussion

JBS is a rare autosomal recessive disorder caused by a mutation in the *URB1 *gene. It was first described in 1971, and less than 100 cases have been reported since then [2,8]. To the best of our knowledge, our patient was the first case of JBS in Bahrain.

**Figure 7:**
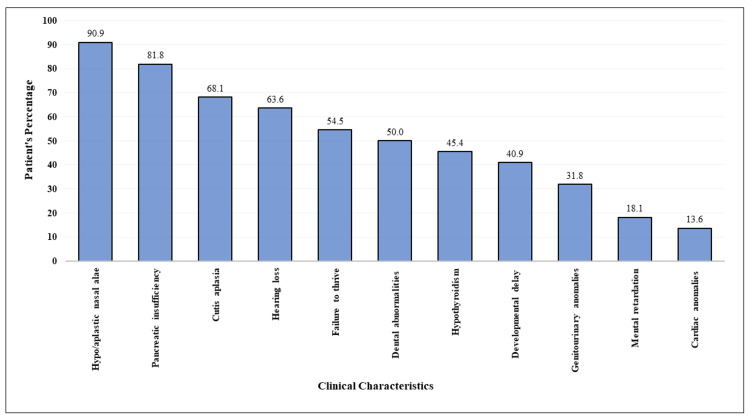
Clinical characteristics of patients with Johanson-Blizzard syndrome as reported in the literature. Summary of 21 previously reported cases along with our patient [4,10-24].

This syndrome affects many systems and causes a wide range of clinical abnormalities. However, the most prominent feature is exocrine pancreatic insufficiency [6]. Upon literature review, pancreatic insufficiency presents in nearly all JBS cases (Figure 7) [4,10-24]. 

Nonetheless, one patient without pancreatic insufficiency was reported by Corona-Rivera et al. [16]. Although pancreatic insufficiency is present in many conditions, it is considered a challenge for pediatricians to differentiate JBS from other syndromes with pancreatic insufficiency [14]. The occurrence of steatorrhea soon after birth was suggestive of pancreatic insufficiency despite an initial normal fecal tryptic activity. Subsequently, she was started on pancreatic enzyme replacement therapy.

Other characteristic abnormalities, including hypoplastic or aplastic nasal alae, cutis aplasia on the scalp, hearing loss, hypothyroidism, developmental delay, failure to thrive, dental abnormalities, mental retardation, cardiac anomalies, and genitourinary anomalies have been reported [6]. Similarly, hypoplastic alae nasi and cutis aplasia on the scalp were found in our patient. Moreover, microcephaly and mild hypotelorism were also detected. Subsequently, she was found to have delayed developmental milestones, hypotonia, subclinical hypothyroidism, bilateral sensorineural deafness, dental anomalies, and ocular manifestations, but she had fair intellectual function. Oblique facial clefts, and hepatic, hematological, and ocular manifestations, which are less commonly reported abnormalities in JBS, were not detected in our patient [16,17,21].

The diagnosis of JBS is usually made during infancy or childhood by the presence of JBS characteristic phenotypic features, laboratory findings, radiological imaging, and genetic testing [10,13,14,17]. Our patient was found to have the characteristic phenotypic features of JBS. Thus, laboratory investigations were requested for her, including stool fat globules, tryptic activity, and thyroid function tests. Results showed features of pancreatic insufficiency and subclinical hypothyroidism. Other laboratory tests such as serum amylase, lipase, a low level of trypsinogen, fat-soluble vitamins, and stool examination are guidance tools for detecting pancreatic insufficiency [14,22]. In addition, thyroid function tests are usually requested to diagnose hypothyroidism [4,17].

In terms of radiological imaging, the antenatal ultrasound assessment of our patient showed an abnormal shape of the nose, suggestive of absent alae nasi. This finding has not been reported in any previous studies. An abdominal CT scan is usually requested to look for fatty tissue replacement in the pancreas, which indicates exocrine pancreatic insufficiency [14,22]. As for our patient, abdominal US was requested instead of abdominal CT, and it was normal. Thus, the diagnosis of pancreatic insufficiency was based on laboratory investigations. In addition to its role in diagnosing pancreatic-related conditions, a CT scan is also used to detect otologic abnormalities, nasal alae abnormalities, and ocular manifestations [12,14,21]. Accordingly, brain CT and MRI were performed for our patient and resulted in a diagnosis of cochleovestibular anomalies. Bilateral sensorineural deafness was confirmed by audiological testing and was managed with bilateral cochlear implantation. The echocardiogram was unremarkable for our patient. Yet, cardiac lesions have been previously reported in patients with JBS. Alwadee et al. [12] reported patent ductus arteriosus, while Almashraki et al. reported a small atrial septal defect [4].

The diagnosis of JBS in our patient was also confirmed by a genetic test that showed a novel sequence change mutation of the UBR1 gene (1 bp duplication causing a frameshift). The *UBR1 *gene plays a key role in the development or maintenance of acinar cells, and defects in this gene result in exocrine pancreatic dysfunction [1]. According to Sukalo et al., 59 mutations are known to be related to JBS, with unrepeated gene mutations running in families; however, the most recorded gene mutations are missense mutations [25].

The treatment of JBS depends on symptomatic management [8]. As for our patient, a multidisciplinary approach was needed to achieve a better outcome. At first, cochlear implantation was performed for the treatment of sensorineural deafness. Accordingly, our patient showed significant improvement in her auditory skills, speech, and language development, which helped achieve a reasonable educational outcome. In addition, speech therapy sessions were required for her. Alwadee et al. highlighted the need for early diagnosis of hearing impairment and the importance of earlier cochlear implantation in JBS patients to improve patient outcomes [12].

Our patient had nasal reconstructive plastic surgery, a dacryocystorhinostomy, and dental procedures. To the best of our knowledge, plastic surgery and dacryocystorhinostomy have not been reported in any previous JBS patient. Nonetheless, Santhosh et al. mentioned the use of a removable partial denture in the process of treating JBS patients, which had a favorable effect on the patient [19].

Our patient had a good outcome. Besides fair intellectual function, she has an average school performance and satisfactory communication skills, which shows the importance of the multidisciplinary approach in this group of patients.

## Conclusions

JBS is a very rare genetic syndrome. Thus, we reported the first case of JBS in Bahrain with a novel mutation. An antenatal assessment might be useful in detecting the associated nasal abnormalities in this syndrome. In addition, treatment of pancreatic insufficiency, early detection of hearing impairment, and reconstructive nasal surgeries might result in significant improvement in patient's condition. Therefore, this report highlights the importance of the early diagnosis and the vital role of the multidisciplinary approach in reaching the best possible outcome. Further studies focusing on the quality of life and long-term outcome of this syndrome are still needed.
